# Lcapy: symbolic linear circuit analysis with Python

**DOI:** 10.7717/peerj-cs.875

**Published:** 2022-02-18

**Authors:** Michael Hayes

**Affiliations:** Electrical and Computer Engineering, University of Canterbury, Christchchurch, New Zealand

**Keywords:** Linear circuit analysis, Symbolic computation, Python

## Abstract

Lcapy is an open-source Python package for solving linear circuits symbolically. It uses a superposition of DC analysis, AC (phasor) analysis, transient (Laplace) analysis, and noise analysis. Expressions are evaluated using the computer algebra system SymPy. Lcapy can model circuits comprised of combinations of one-port and two-port networks or circuits specified using a netlist with a Spice-like notation. Lcapy can present the system of equations produced from nodal analysis, modified nodal analysis, loop analysis, and state-space analysis. Expressions can be formatted into many representations, parameterized, and transformed to other domains. Dimensional analysis is performed to reduce user errors and to present results with units. Both continuous and discrete signals are supported. Lcapy produces high-quality output. Textbook quality schematics in a number of different formats can be generated from netlists and customized for different conventions. Expressions can be formatted into LaTeX format for inclusion into a document or numerically evaluated and plotted. An overview of the features and capabilities of Lcapy is presented, along with implementation details and performance considerations.

## Introduction

Lcapy is an open-source Python tool to symbolically generate and solve the system of integro-differential equations for a linear electrical, mechanical, or electro-mechanical circuit.

Unlike most other circuit analysis programs, Lcapy stores all values and expressions symbolically. It utilizes the powerful Python symbolic mathematics package, Sympy (Joyner et al., 2012; Meurer et al., 2017). The primary advantage of symbolic analysis is that it provides an exact solution and avoids the trial-and-error nature of system analysis using numerical simulation. Moreover, it avoids the instability and inaccuracy associated with numerical integration required by time-stepping simulation. The symbolic solution provides insight into system behavior, for example, how a component influences the poles of a power-electronics controller (Heffernan, Mitchell & Hayes, 2020). Using symbolic mathematical tools allows expressions to be easily converted to standard forms, such a pole-zero-gain or partial fraction, to gain further insight into system behaviour.

Lcapy started in 2013 as a collection of Python classes for handling rational functions for modelling piezo-electric transducers. This was augmented with a collection of component classes that could be organised into networks using parallel and series operators (Hayes, 2014). As components were added, the network was represented either as a Thévenin or Norton model. This was efficient since the network could be represented as a Laplace-domain generalized impedance with either a current source or a voltage source. Later, networks were stored as an abstract syntax tree to provide a more general representation. Netlists were then added to describe the component connectivity but analysis could only be performed in the Laplace domain. Analysis of networks was performed by converting them to netlists. Lcapy was then largely rewritten to represent voltages and currents as a superposition of DC, AC, transient, and noise signals. Thus, the response for arbitrary sources could be analysed. Many other features have since been added; such as semi-automated textbook-quality schematic generation (Hayes, 2016), discrete-time analysis, plotting, expression manipulation, network synthesis, and unit tracking.

Open-source tools are available for numerical circuit simulation, including SPICE (Nagel & Pederson, 1973) and its many derivatives, such as PySpice (https://pypi.org/project/PySpice/), and Qucs (Brinson & Jahn, 2009), an enhancement to SPICE. These tools are excellent for the generation of numerical solutions to general, non-linear circuits. However, they do not provide the insight of symbolic analysis.

A number of symbolic circuit analysis packages have been developed, primarily through the 1980s and 1990s (Fernández & Rodríguez-Vázquez, 1996). These include: ISAAC (Interactive symbolic analysis of analog circuits), a common Lisp program for integrated circuit design that uses compacted modified nodal analysis (CMNA) with heuristics to reduce the number of symbolic term cancellations (Walscharts, Gielen & Sansen, 1989; Gielen, Walscharts & Sansen, 1989); ASAP (analog symbolic analysis program), a C program that expands transistors in SPICE netlist using library models for small-signal symbolic analysis (Fernández, Rodríguez-Vázquez & Huertas, 1990); Sspice, a C program for small-signal analysis that uses biasing information to simplify the transfer function (Wierzba et al., 1989; Srivastava, Wierzba & MacKay, 1990); AnalogSifter, designed for large integrated circuits using approximate transfer functions (Hsu & Sechen, 1993, 1994); SNAP (Symbolic and Numerical Analysis Program), a Windows program that linearises non-linear circuits around an operating point and can approximate the symbolic analysis (Biolek, 2000; Kolka, Biolek & Biolkova, 2008); SCIASCA, a Visual Java++/Maple program using nullor-based models (Tlelo-Cuautle et al., 2004); SCAM (https://lpsa.swarthmore.edu/Systems/Electrical/mna/MNA6.html) (symbolic circuit analysis in MATLAB); Akhab (https://ahkab.github.io/ahkab), a Python program with a SPICE-like command sequence; Sapwin/SapecNG/QSapecNG, Windows-based C++ programs (Grasso et al., 2014); MSCAM (modified symbolic circuit analysis in MATLAB) (Ushie, Abbod & Ashigwuike, 2015); AICE (analog IC explorer), a C++ program with web-based interface that uses advanced graph techniques (Shi, 2017); CircuitNav (https://circuitnav.pythonanywhere.com/), a web-based application for generating a symbolic system of equations given a netlist, and SliCAP (https://www.analog-electronics.eu/slicap/slicap.html) (Symbolic Linear Circuit Analysis Program), a Python (previously MATLAB) program for teaching circuit analysis.

There is also an unnamed MATLAB toolbox that uses transfer function approximation (Vazzana, Grasso & Pennisi, 2017) and another unnamed tool that uses a graph reduction decision diagram (GRDD) algorithm (Chen & Shi, 2006). In addition, a summary of some older programs is given by Fernández & Rodríguez-Vázquez (1996).

Most of these tools are are designed specifically for integrated circuit analysis (Hsu & Sechen, 1993), are tied to a particular operating system, and are no longer available. A possible reason is that most are not open-source (Huelsman, 1996) and thus have not been maintained over time.

A novel aspect of Lcapy is that it decomposes signals into a number of transform domains (DC, phasor, Laplace), performs modified nodal analysis for each domain, and superimposes the result. A rich variety of transformations is provided to convert between the various domains and to present results in different formats. Lcapy uses an object-oriented approach and provides a comprehensive selection of expression classes to specify the transform domain and data quantity.

This paper is not a comprehensive description of the capabilities of Lcapy (this can be found in the supporting documentation (https://lcapy.readthedocs.io) (Hayes, 2020)). Instead, the focus is on the philosophy, use cases, and implementation. The paper starts with a brief overview in “Overview”, the major use cases are described with some examples in “Basic usage”, circuit analysis techniques are described in “Circuit Analysis”, additional features are presented in “Additional Features”, details of the implementation are discussed in “Implementation Details”, and a performance analysis is shown in “Performance”. The paper concludes with a discussion and conclusions in “Discussion” and “Conclusions and Future Work”.

## Overview

Lcapy is an open-source Python package for symbolic linear circuit analysis. Python was chosen since it is platform independent (van Rossum & Drake, 1995), can be used with a command-line interface or an integrated development environment, and has excellent packages for plotting—Matplotlib, symbolic analysis—Sympy (Joyner et al., 2012; Meurer et al., 2017), and numerical analysis—NumPy (Oliphant, 2006; van der Walt, Colbert & Varoquaux, 2011) and SciPy (Virtanen et al., 2020). It is also well documented with online support.

The author has used Lcapy for modelling low-noise operational amplifier circuits, designing analog and digital filters, simulating polyphase power systems, fitting models to electrode impedance data for electromagnetic flowmeters, designing controllers for power-electronics, modelling impedance compensation systems for biomedical instrumentation, and modelling piezoelectric transducers. He and others have also used Lcapy for producing textbook quality schematics, generating exam questions, checking exam and homework answers, and teaching students circuit theory.

An object-oriented design was chosen to simplify both the implementation and user interface. The classes provided by Lcapy range from simple expressions, to one-port and two-port networks, to complete netlist models. The object oriented approach makes available methods for a given object clear to the user. For example, time-domain objects have a Laplace transform method but Laplace-domain objects have an inverse Laplace transform method.

Lcapy uses an instance-based netlist similar to SPICE (Nagel & Pederson, 1973) to describe how components are connected. It is simply a list of named components, each with named nodes, see Listing 1. Circuit analysis is performed by converting the netlist into sub-netlists; one for each of the DC, AC, transient, and noise domains identified from the independent sources. Each of the sub-netlists are solved independently using modified nodal analysis and the results are stored as a superposition of DC, AC, transient, and noise signals. These superpositions can then be transformed to the time domain or Laplace domain as required by the user.

**Listing 1:**
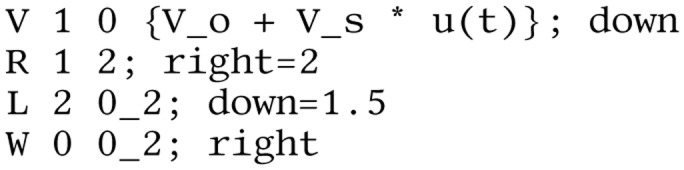
Lcapy netlist for V-R-L circuit. Note, drawing options are specified after the semicolon.

As well as electrical components, Lcapy netlists can contain masses, springs, and dampers for modelling mechanical or electro-mechanical networks. The mechanical analogue II (impedance analogue) is employed where voltage is equivalent to force and current is equivalent to speed. Thus a mass is analogous to an inductor, a spring is analogous to a capacitor, and a damper is analogous to a resistor.

## Basic usage

Lcapy has three major use cases: network and circuit analysis using combinations of one-port and two-port models; network and circuit analysis using netlists; and symbolic expression manipulation for interpretation of results. These are discussed in the following sections.

### Expressions

Lcapy has a number of pre-defined variables associated with different domains: f for the Fourier (frequency) domain, F for the normalized Fourier domain, s for the Laplace (s) domain, t for the time domain, omega for the angular Fourier domain, and Omega for the normalized angular Fourier domain. These variables can be utilised to create expressions or to specify the result domain for transformations, for example^1^:

>>> from lcapy import s, t, exp

>>> H = s * (s + 2)/ (s + 3) * exp(-2 * s)

>>> H(t)

$3{e^{6 - 3t}}u\left( {t - 2} \right) - \delta \left( {t - 2} \right) + {\delta ^{\left( 1 \right)}}\left( {t - 2} \right)\;\;{\rm for}\,t \ge 0.$

Here *δ*(*t*) is the Dirac delta and *δ*^(1)^(*t*) is the first derivative of the Dirac delta. As a consequence of the unilateral Laplace transform, the result is only known for *t ≥* 0. However, if the result is known to be zero for *t* < 0, this can be specified by asserting the causal argument, for example:

>>> from lcapy import s, t, exp

>>> H = s * (s + 2) / (s + 3) * exp(-2 * s)

>>> H(t, causal = True)

$3{e^{6 - 3t}}u\left( {t - 2} \right) - \delta \left( {t - 2} \right) + {\delta ^{\left( 1 \right)}}\left( {t - 2} \right).$

Lcapy can display expressions in a number of forms. In each case the expressions are factored into a rational function and an optional exponential function (the latter is used to support Laplace-domain delays):

(1) $$H(s) = \displaystyle{{N(s)} \over {D(s)}}\exp ( - sT).$$

Rational functions can be represented in many forms. Table 1 shows the representations that Lcapy supports.

**Table 1:** Rational function representation methods.

Method	Example	Description
general()	$\displaystyle{{5{s^2} + 5} \over {2{s^2} + 9s + 4}}$	Numerator and denominator are polynomials with no factoring of coefficients.
standard()	$\displaystyle{{ - 45s - 10} \over {4{s^2} + 18s + 8}} + \displaystyle{5 \over 2}$	Sum of a polynomial and a strictly rational function.
canonical()	$\displaystyle{{\displaystyle{{5{s^2}} \over 2} + \displaystyle{5 \over 2}} \over {{s^2} + \displaystyle{{9s} \over 2} + 2}}$	Denominator is monic (default).
expandcanonical()	$\displaystyle{{5{s^2}} \over {2{s^2} + 9s + 4}} + \displaystyle{5 \over {2{s^2} + 9s + 4}}$	Sum of rational functions where the numerator of each term contains a unique monomial.
ZPK()	$\displaystyle{{5\left( {s - {\rm j}} \right)\left( {s + {\rm j}} \right)} \over {2\left( {s + \displaystyle{1 \over 2}} \right)\left( {s + 4} \right)}}$	Zero-pole-gain or factored form where the numerator and denominator are factored.
partfrac()	$\displaystyle{5 \over 2} - \displaystyle{{85} \over {7\left( {s + 4} \right)}} + \displaystyle{{25} \over {28\left( {s + \displaystyle{1 \over 2}} \right)}}$	Partial fraction form.
recippartfrac()	$\displaystyle{5 \over 4} - \displaystyle{{25} \over {7\left( {2 + \displaystyle{1 \over s}} \right)}} + \displaystyle{{85} \over {112\left( {\displaystyle{1 \over 4} + \displaystyle{1 \over s}} \right)}}$	Partial fraction form using the reciprocal of the variable.
timeconst()	$\displaystyle{{\displaystyle{{5{s^2}} \over 4} + \displaystyle{5 \over 4}} \over {\displaystyle{{{s^2}} \over 2} + \displaystyle{{9s} \over 4} + 1}}$	Numerator and denominator are factored as time-constants.

Some of the other methods that can be applied to Laplace-domain expressions include: initial_value(), final_value(), differentiate(), integrate(), delay(), zeros(), poles(), residues(), transient_response(), frequency_response(), and step_response(). For example:

>>> from lcapy import s

>>> V = s**3 / ((s + 3) * (s + 4))

>>> V.poles()

$\left\{ { - 3:1,\; - 4:1} \right\}.$

 This dictionary indicates a pair of real poles, each with a single occurrence.

### Discrete-time expressions

Lcapy also has a number of pre-defined variables associated with discrete-time signals: k for the discrete-frequency sample index, n for the discrete-time sample index, and z for the z-transform domain. These can be used to compose discrete-time signals, for example:

>>> from lcapy import delta, n

>>> x = delta(n) + 2 * delta(n - 2)

>>> x.seq()

$\left[ {1,\;0,\;2} \right]$

and

>>> from lcapy import delta, n, z

>>> x = delta(n) + 2 * delta(n - 2)

>>> x(z)

$1 + \displaystyle{2 \over {{z^2}}}.$

### Sequences

Discrete-time, discrete-Fourier, and Z-domain expressions can be converted to and from sequences. These can be convolved, shifted, transformed, *etc*. For example,

>>> from lcapy import symbols, delta, n

>>> a, b = symbols(’a b’)

>>> x = a * delta(n - 1) + b * delta(n - 2)

>>> x.seq()

$\left\{ {\underline 0 ,a,b} \right\},$

where the underscore marks the origin. Similarly, sequences can be converted to expressions:

 >>> seq((1, 2, 3, ’a’)).expr

$a\delta \left[ {n - 3} \right] + \delta \left[ n \right] + 3\delta \left[ {n - 2} \right] + 2\delta \left[ {n - 1} \right].$

### One-port networks

Networks can be constructed by connecting one-port (two-terminal) and two-port components. Basic one-port components include: Vdc (DC voltage source), Idc (DC current source), Vac (AC voltage source), Iac (AC current source), R, G, C, and L. These are augmented by V (generic voltage source), I (generic current source), Y (generic admittance), and Z (generic impedance). More esoteric components include gyrators and constant phase elements.

Here is an example that combines an 8 V source in series with a parallel combination of 6 ohm and 3 ohm resistors:

>>> from lcapy import V, R

>>> net = V(8) + (R(6) | R(3))

>>> net

$V(8) + (R(6)|R(3)).$

Note, Lcapy overloads the Python | operator to denote a parallel operation. Also note that when the net object is printed, the underlying components forming the network are shown. This is achieved using an abstract syntax tree to represent the component connections.

A network can be simplified:

>>> net.simplify()

$V(8) + R(2)$

and transformed into its Norton or Thévenin equivalent:

>>> net.norton()

$G(1/2)|I(4).$

Attributes of one-port networks include: Isc—short-circuit current; Voc—open-circuit voltage; Y—generalized admittance; and Z—generalized impedance.

### Two-port networks

**Figure 1:**
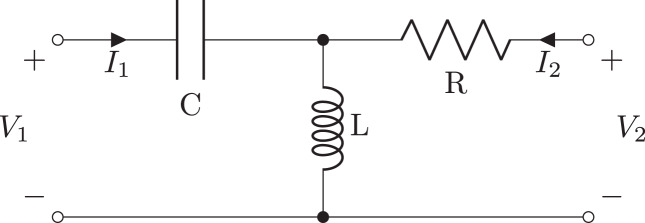
Schematic of second order filter example generated as a two-port network, using a T-Section or by chaining C, L, and R oneport components.

Two-port networks can be created from one-port components using ladder descriptions (Khanshan (2007)), primitive two-ports (Shunt, Series, LSection, TSection, TwinTSection, BridgedTSection, PiSection, Ladder, GeneralTxLine, LosslessTxLine, TxLine), or by combinations of two-ports using series(), parallel(), and chain() methods. Furthermore, they can be generated from a netlist. For example, the filter shown in Figure 1 can be represented as a two-port using:

>>> from lcapy import TSection, L, C, R

>>> a = TSection(C(’C’), L(’L’), R(’R’))

The same network can be constructed by chaining series and shunt elements:

>>> from lcapy import Series, Shunt, L, C, R

>>> a = Series(C(’C’)).chain(Shunt(L(’L’))).chain(Series(R(’R’)))

A two-port object has methods for generating A (chain) parameters, B (inverse chain) parameters, G (inverse hybrid) parameters, H (hybrid) parameters, S (scattering) parameters, T (transmission) parameters, Y (admittance) parameters, and Z (impedance) parameters (Hayt, Kemmerly & Durbin, 2006). For the above example, the Z parameters can be found using:

>>> a.Zparams

$\left[ {\matrix{ { - Ls\left( { - 1 - \displaystyle{1 \over {CL{s^2}}}} \right)} & {Ls} \cr {Ls} & { - Ls\left( { - 1 - \displaystyle{R \over {Ls}}} \right)} \cr } } \right].$

A unique feature of Lcapy is polyphase two-port networks. These use block-matrix forms for the parameter matrices and are useful for modelling power systems. They have methods for converting to symmetrical sequence components.

### Netlists

**Figure 2:**
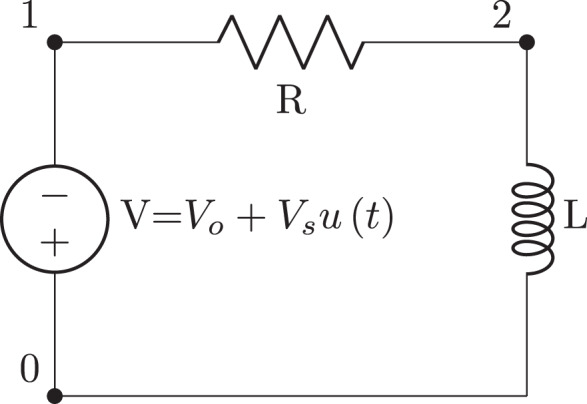
Simple V-R-L circuit schematic generated from the netlist in Listing 1.

More complex circuit topologies are best described using netlists. The Lcapy netlist syntax is similar to that of SPICE. Usually nodes are numbered, but they can have symbolic names. The netlist can be loaded from a file or generated incrementally using the add method of the Circuit class. For example, Listing 1 describes the circuit shown in Fig. 2.

A Circuit object is collection of components and nodes. The nodes are accessed using Python’s array-indexing notation, for example,

>>> cct[2] - cct[’a7’]

Nodes have a number of attributes including: V—the voltage between the node and ground; dpY—the driving-point generalized admittance between the node and ground; dpZ—the driving-point generalized impedance between the node and ground. Thus the potential of node 2 with respect to ground can be found using:

>>> cct[2].V

Components are dynamically added as attributes of the Circuit class. Thus components can be accessed using the Python’s attribute notation. For example:

>>> cct.C1

Each component has several attributes as shown in Table 2. For example, the current through the inductor L1 in the netlist described by Listing 1 is found using:

**Table 2:** Component attributes.

Attribute	Description
V	Voltage across the component
I	Current through the component
Voc	Voltage across the component when open-circuited
Isc	Current through the component when short-circuited
Y	Generalized admittance of the component
Z	Generalized impedance of the component
dpY	Driving-point generalized admittance across the component
dpZ	Driving-point generalized impedance across the component

>>> from lcapy import Circuit

>>> cct = Circuit(’VRL1.sch’)

>>> cct.L1.I

$\left\{ {{\rm dc}:\displaystyle{{{V_o}} \over R},\;{\rm s}:\displaystyle{{\displaystyle{1 \over L}{V_s}} \over {{s^2} + \displaystyle{{Rs} \over L}}}} \right\}.$

The output is a dictionary showing the superposition of a DC component and a transient-component described in the Laplace domain.

The time-domain response can be found using Python’s call notation:

>>> cct.L1.I(t)

This transforms each component of the superposition into the time domain and sums the result to give:

${V_s}\left( {\displaystyle{1 \over R} - \displaystyle{{{e^{ - {{Rt} \over L}}}} \over R}} \right)u\left( t \right) + \displaystyle{{{V_o}} \over R}.$

Similarly, the total Laplace-domain response can be found using:

>>> cct.L1.I(s)

$\displaystyle{{\displaystyle{1 \over L}{V_s}} \over {{s^2} + \displaystyle{{Rs} \over L}}} + \displaystyle{{{V_o}} \over {Rs}}.$

**Figure 3:**
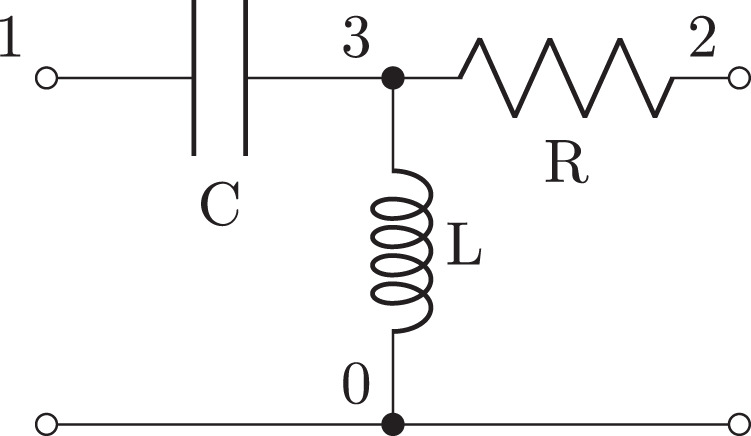
Schematic of second order filter example generated from the netlist in Listing 2.

A common circuit analysis problem is to find the transfer function between two pairs of nodes. There are several ways this can be achieved with Lcapy. For example, consider the filter network shown in Fig. 3. First, the independent sources need to be killed and an arbitrary Laplace-domain voltage source connected across the input nodes. The transfer function is then found from the ratio of the voltage measured across the output nodes to the input voltage. An alternative approach is to use the transfer() method which implicitly kills any independent sources. For example:

**Listing 2:**
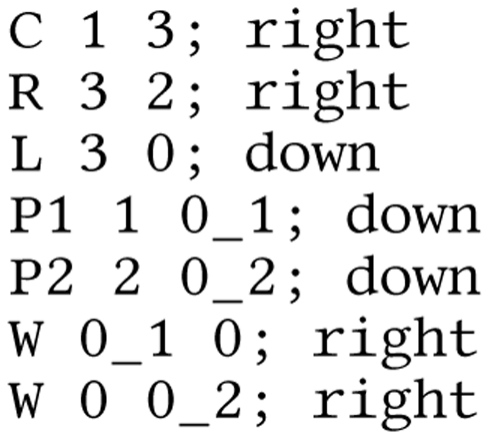
Lcapy netlist description of a second-order R-L-C filter. The W (wire) and P (port) components are purely for drawing.

>>> H = cct.transfer(1, 0, 2, 0)

>>> H.canonical()

$\displaystyle{{{s^2}} \over {{s^2} + \displaystyle{1 \over {CL}}}}.$

A more general approach is to generate a two-port model. For example, the Z-parameters can be found using:

>>> tp = cct.twoport(1, 0, 2, 0)

>>> tp.Zparams

$\left[ {\matrix{ {Ls + \displaystyle{1 \over {Cs}}} & {Ls} \cr {Ls} & {Ls + R} \cr } } \right].$

The common two-port models are supported, including the A, B, G, H, S, T, Y, and Z-parameter models (Hayt, Kemmerly & Durbin, 2006). They can be converted interchangeably provided the matrices are not singular.

Other methods of the Circuit class are shown in Table 3. These are augmented with methods for manipulating a netlist as listed in Table 4.

**Table 3:** Circuit methods.

Method	Description
describe()	Prints how the circuit is analysed
thevenin()	Returns a Thevenin object between the two specified nodes
norton()	Returns a Norton object between the two specified nodes
twoport()	Returns a TwoPort object specified by two pairs of nodes
Voc()	Returns the open-circuit voltage between two specified nodes
Isc()	Returns the short-circuit current between two specified nodes
Y()	Returns the generalized admittance between specified nodes (with the independent sources killed)
Z ()	Returns the generalized impedance between specified nodes (with the independent sources killed)

**Table 4:** Circuit manipulation methods.

Method	Description
ac()	Creates a circuit using only the AC independent sources
dc ()	Creates a circuit using only the DC independent sources
transient ()	Creates a circuit using only the transient independent sources
simplify ()	Combines components in series or parallel to simplify the netlist
kill ()	Kills specified independent sources (voltage sources become short-circuits and current sources become open-circuits)
kill_except ()	Kills all but the specified independent sources
s_model ()	Converts sources to the Laplace-domain and represents reactive components as impedances
state_space_model()	Creates a state-space model by replacing inductors with current sources and capacitors with voltage sources
r_model ()	Creates a resistive equivalent model using companion circuits for time-stepping simulation
noise_model ()	Replaces resistors with a series combination of a resistor and a noise voltage source
subs()	Substitutes symbolic component values

## Circuit analysis

**Figure 4:**
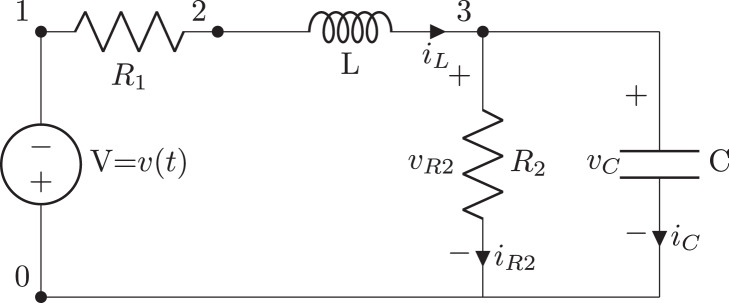
State-space example circuit.

As well as calculating node voltages and branch currents, Lcapy can also display the equations for nodal analysis, modified nodal analysis, mesh analysis, and state-space analysis. For the following, consider the circuit shown in Fig. 4 defined by the netlist in Listing 3.

**Listing 3:**
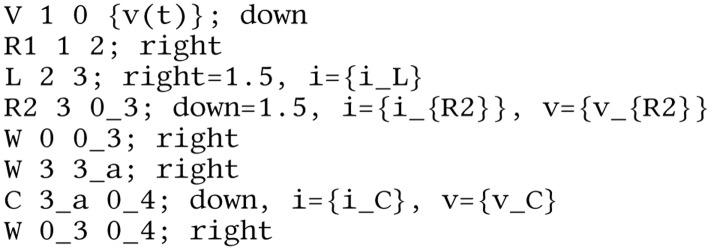
Lcapy netlist for the circuit analysis example of Fig. 4.

### Nodal analysis

Lcapy can output the nodal equations by applying Kirchhoff’s current law at each node in a circuit. For example:

>>> from lcapy import Circuit

>>> cct = Circuit(’example1.sch’)

>>> na = cct.nodal_analysis()

>>> na.nodal_equations()

$\left\{ {} \right.{\rm 1}:\;{v_1}(t) = v(t),$

${\rm 2}:\;\displaystyle{{ - {v_1}(t) + {v_2}(t)} \over {{R_1}}} + \displaystyle{{\int\limits_{ - \infty }^t \left( {{v_2}(\tau ) - {v_3}(\tau )} \right){\kern 1pt} d\tau } \over L} = 0,$

${\rm 3}:\;C\displaystyle{{\rm d} \over {\rm dt}}{v_3}(t) + \displaystyle{{{v_3}(t)} \over {{R_2}}} + \displaystyle{{\int\limits_{ - \infty }^t \left( { - {v_2}(\tau ) + {v_3}(\tau )} \right){\kern 1pt} d\tau } \over L} = 0\left. {} \right\}$

This is a dictionary keyed by the circuit node names.

The nodal equations can be formulated in the Laplace-domain using the laplace() method to convert the circuit. For example:

>>> na = cct.laplace().nodal_analysis()

>>> na.nodal_equations()

$\left\{ {} \right.{\rm 1}:\;{V_1}(s) = V(s),$

${\rm 2}:\;\displaystyle{{ - {V_1}(s) + {V_2}(s)} \over {{R_1}}} + \displaystyle{{{V_2}(s) - {V_3}(s)} \over {Ls}} = 0,$

${\rm 3}:\;Cs{V_3}(s) + \displaystyle{{{V_3}(s)} \over {{R_2}}} + \displaystyle{{ - {V_2}(s) + {V_3}(s)} \over {Ls}} = 0\left. {} \right\}.$

The matrix formulation can also be generated for the Laplace-domain, DC, and phasors. For example:

>>> na = cct.laplace().nodal_analysis()

>>> na.matrix_equations()

$\left[ {\matrix{ {{V_1}(s)} \cr {{V_2}(s)} \cr {{V_3}(s)} \cr } } \right] = {\left( {\left[ {\matrix{ 1  0  0  \cr { - \displaystyle{1 \over {{R_1}}}}  {\displaystyle{1 \over {{R_1}}} + \displaystyle{1 \over {{Ls}}}}  { - \displaystyle{1 \over {{Ls}}}}  \cr 0  { - \displaystyle{1 \over {{Ls}}}}  {Cl{_{{\rm aplace}}} + \displaystyle{1 \over {{R_2}}} + \displaystyle{1 \over {{Ls}}}} \cr } } \right]} \right)^{ - 1}}\left[ {\matrix{ {V(s)} \cr 0 \cr 0 \cr } } \right].$

### Mesh analysis

Lcapy can output the mesh equations by applying Kirchhoff’s voltage law around each mesh loop in a circuit. For example:

>>> from lcapy import Circuit

>>> cct = Circuit(‘example1.sch’)

>>> la = cct.loop_analysis()

>>> la.mesh_equations()

$\left\{ {i_1}(t):\;L\displaystyle{{\rm d} \over {\rm d}t}\left( { - {i_1}(t)} \right) - {R_1}{i_1}(t) + {R_2}\left( { - {i_1}(t) + {i_2}(t)} \right) + v(t) = 0,\right.$

$ {i_2}(t):\;{R_2}\left( { - {i_1}(t) + {i_2}(t)} \right) + \displaystyle{{\int\limits_{ - \infty }^t \left( { - {i_1}(\tau ) + {i_2}(\tau )} \right){\kern 1pt} d\tau } \over C} = 0 {} \Bigg\}$

This is a dictionary indexed by the mesh current.

### State-space analysis

State-space analysis is performed by assigning capacitor voltages and inductor currents as state variables. The state equations are found using:

>>> from lcapy import Circuit

>>> cct = Circuit(’example1.sch’).

>>> ss = cct.state_space()

>>> ss.state_equations()

$\left[ {\matrix{ {\displaystyle{{\rm d} \over {\rm d}t}{i_L}(t)}  \cr {\displaystyle{{\rm d} \over {\rm d}t}{v_C}(t)} \cr } } \right] = \left[ {\matrix{ {\displaystyle{1 \over L}}  \cr 0 \cr } } \right]\left[ {\matrix{ {v(t)} \cr } } \right] + \left[ {\matrix{ { - \displaystyle{{{R_1}} \over L}}  { - \displaystyle{1 \over L}}  \cr {\displaystyle{1 \over C}}  { - \displaystyle{1 \over {C{R_2}}}} \cr } } \right]\left[ {\matrix{ {{i_L}(t)} \cr {{v_C}(t)} \cr } } \right].$

Similarly, the output equations are found using:

>>> ss.output_equations()

$\left[ {\matrix{ {{v_1}(t)} \cr {{v_2}(t)} \cr {{v_3}(t)} \cr } } \right] = \left[ {\matrix{ 1 \cr 1 \cr 0 \cr } } \right]\left[ {\matrix{ {v(t)} \cr } } \right] + \left[ {\matrix{ 0 & 0 \cr { - {R_1}} & 0 \cr 0 & 1 \cr } } \right]\left[ {\matrix{ {{i_L}(t)} \cr {{v_C}(t)} \cr } } \right].$

The state-space analyser can generate state-transition matrices, system transfer functions, system impulse responses, and the characteristic polynomial. For example, the characteristic polynomial for the circuit in Fig. 4 is generated by:

>>> ss.P

$\displaystyle{{{R_2} + \left( {Ls + {R_1}} \right)\left( {C{R_2}s + 1} \right)} \over {CL{R_2}}}.$

### Initial value problems

If any capacitor or inductor in a netlist has an initial value, the transient response is calculated as an initial value problem using Laplace transforms. The result is only valid for *t* ≥ 0.

### Simulation

Lcapy can numerically simulate a circuit using time-stepping numerical integration. Both the trapezoidal and backward-Euler integrators are provided. Here is an example of use:

>>> from lcapy import Circuit

>>> from numpy import linspace

>>> cct = Circuit(" " "

… V 1 0 {10 * u(t)}; down

… R 1 2 5; right

… C 2 3 0.1; right

… L 3 0_3 0.5; down

… W 0 0_3; right" " ")

>>> tv = linspace(0, 1, 100)

>>> results = cct.sim(tv)

>>> ax = cct.R.v.plot(tv, label = ’analytic’)

>>> ax.plot(tv, results.R.v, ’C1–’, label=’simulated’)

>>> ax.legend()

**Figure 5:**
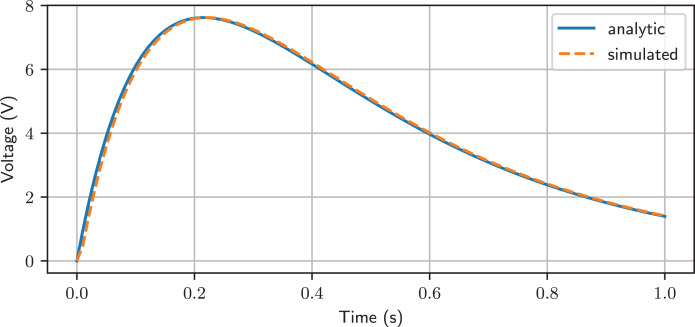
Comparison between simulated and analytic result.

In this example, the sim() method takes a NumPy array of time values to numerically evaluate the circuit at. The variable results is a SimulationResults object. This can be indexed by node name. It also has an attribute for each circuit component; for example, results.R1.v is a NumPy array for the time-varying voltage across R1. The plotted result is shown in Fig. 5.

## Additional features

### Network synthesis

Networks can be created using network synthesis techniques given an impedance or admittance expression. For example:

>>> from lcapy import s, Impedance

>>> Z = Impedance((4 * s**2 + 3 * s + ’1 / 6’) / (s**2 + 2 * s / 3))

>>> Z.network()

$((C(1) + R(2))|C(3)) + R(4).$

Note, in this example the ratio 1/6 is placed in quotes to prevent Python converting the result to a floating point number before Lcapy converts it to a rational number. This overcomes an unfortunate side-effect of the Python parser.

Lcapy provides many network synthesis methods, including recognising the common parallel/serial combinations of resistors, inductors, and capacitors plus more generic methods such as Foster I, II and Cauer I, II (Bakshi & Bakshi, 2005).

### Random networks

Networks with a random topology can generated with the random_network() function. This is useful for automated exam question generation. For example:

>>> from lcapy import random_network

>>> net = random_network(num_resistors = 4, num_voltage_sources = 1, kind = ’dc’)

This example generates a DC network with four resistors and one voltage source. The kind argument can be ac, dc, or transient. The number of parallel connections can be specified with the num_parallel argument.

### Parameterization

Lcapy can parameterize a number of first-order, second-order, and third-order Laplace-domain expressions. For example:

>>> from lcapy import s

>>> H2 = 3 / (s**2 + 2 * s + 4)

>>> H2p, defs = H2.parameterize()

>>> H2p

$\displaystyle{K \over {\omega _0^2 + 2{\omega _0}s\zeta + {s^2}}},$

where defs is a dictionary of the parameter definitions. In this case the parameter definitions are:

$\left\{ {{\rm K}:3,\;{\rm omega\_0}:2,\;{\rm zeta}:\displaystyle{1 \over 2}} \right\}.$

Using the dictionary of definitions, the original expression can be obtained by substituting the parameter definitions into the parameterized expression.

Systems higher than first-order can be parameterized in different ways. For example:

>>> from lcapy import s

>>> H2 = 3 / (s**2 + 2 * s + 4)

>>> H2p, defs = H2.parameterize(zeta = False)

>>> H2p

$\displaystyle{K \over {\omega _1^2 + {s^2} + 2s{\sigma _1} + \sigma _1^2}}.$

### Polyphase systems

The polyphase module provides classes for handling polyphase signals. This handles conversions between line voltages and currents to phase voltages and currents. In addition, it can decompose line and phase signals into symmetrical components and vice-versa. In each case, the polyphase signals are stored as vectors. An arbitrary number of phases can be supported, although there are many attributes specifically for three-phase systems, such as Vab that returns the phase-phase voltage between the a and b phases.

Here is an example that determines the symmetrical sequence components for three-phase phase-voltages:

>>> from lcapy.polyphase import PhaseVoltageVector

>>> V = PhaseVoltageVector((’Va’, ’Vb’, ’Vc’))

>>> V.sequence()

$\left[ {\matrix{ {\displaystyle{{{V_a}} \over 3} + \displaystyle{{{V_b}} \over 3} + \displaystyle{{{V_c}} \over 3}} \cr {\displaystyle{{{V_a}} \over 3} + \displaystyle{{{V_b}\alpha } \over 3} + \displaystyle{{{V_c}{\alpha ^2}} \over 3}} \cr {\displaystyle{{{V_a}} \over 3} + \displaystyle{{{V_b}{\alpha ^2}} \over 3} + \displaystyle{{{V_c}\alpha } \over 3}} \cr } } \right],$

where *α* = exp(−j2*π*/3) for a three-phase system.

### Printing

Expressions have printing methods similar to Sympy. These are summarised in Table 5^2^. There are other methods for automatic formatting of the expression for a Jupyter notebook (Kluyver et al., 2016) or an IPython interpreter (Pérez & Granger, 2007). These include the use of MathJax (Cervone (2012)) for mathematics formatting in web browsers.

**Table 5:** Expression printing methods.

Method	Description
latex	Formats the expression using LaTeX markup
pretty	Formats the expression using Unicode
pprint	Formats the expression using Unicode if using an interactive terminal otherwise formats the expression using LaTeX markup

### Evaluation

Expressions have an evaluate() method which accepts a NumPy array of values at which to evaluate the expression. Internally, Lcapy uses Sympy’s lambdify() function to convert the expression to executable Python code that can be efficiently evaluated.

### Plotting

Expressions have a plot() method. For Laplace-domain expressions, this is a pole-zero plot. For time-domain expressions, this shows the waveform. In addition, there are specific methods for Bode and Nyquist plots. The plot() methods provide automatic labelling and use the Matplotlib library for high-quality output in a number of formats (Hunter (2007)).

### Schematics

Lcapy can draw circuits specified as networks or netlists. Networks are drawn automatically using a number of layout forms, including as a ladder network. Netlists require drawing hints to describe the component orientation, size, color, *etc*. These hints are used to automatically position the components. LaTeX CircuiTikz (https://www.ctan.org/pkg/circuitikz) macros are output producing text-book quality schematics. Lcapy provides a script, schtex, that can convert a netlist into an image, with a number of formats such as pdf or png. This script can also manipulate the netlist, such as renumbering the nodes.

### Customisation

Lcapy has a config module that can be used for customisation. For example, the printing of $\sqrt { - 1}$ as i or j, aliases for functions such as the Dirac delta or Heaviside unit step, an extensible list of words used for formatting in a Roman font in LaTeX expressions, and which method Sympy uses for matrix inversion.

## Implementation details

Lcapy is implemented using 393 classes within 115 modules^3^. Most modules have a single class but a number, such as Oneport, represent many types of similar one-port component. Lcapy has an additional 170 expression classes that are lazily defined as required.

### Expressions

Expressions are represented using one of the 170 dynamic expression classes^4^. Each of these inherit from a quantity class (voltage, current, impedance, *etc*.) and from a domain class (time, Laplace, Fourier, phasor, discrete-time, *etc*.). Attributes specify the quantity (is_voltage, is_current, *etc*.) and the domain (is_time_domain, is_laplace_domain, *etc*.).

Lcapy expressions are created from factory functions, including: expr() for general expressions, voltage(), current(), impedance(), admittance(), and transfer() (for transfer functions). For example, the expr() factory function chooses one of the expression classes listed in Table 6. Similarly, the voltage() factory function chooses one of the voltage classes, subclassed from a corresponding expression class.

**Table 6:** Expression and sequence domain classes.

Class	Description
AngularFourierDomainExpression	Spectral density as function of angular frequency
AngularFourierNoiseDomainExpression	Noise signal
ConstantDomainExpression	DC signal (or constant value)
DiscreteTimeDomainExpression	Discrete-time signal
DiscreteFourierDomainExpression	Discrete-frequency signal
FourierDomainExpression	Spectral density as function of linear frequency
FourierNoiseDomainExpression	Noise signal
LaplaceDomainExpression	Laplace-domain transformation
NormFourierDomainExpression	Spectral density as function of normalized linear frequency
NormAngularFourierDomainExpression	Spectral density as function of normalized angular frequency
PhasorDomainExpression	AC signal (phasor)
TimeDomainExpression	Time domain signal
SuperpositionExpression	Combination of AC, DC, transient, and noise signals
ZDomainExpression	Z-domain transformation
DiscreteTimeDomainSequence	Discrete-time sequence
DiscreteFourierDomainSequence	Discrete-frequency sequence
ZDomainSequence	Z-domain sequence

There are two special superposition classes: SuperpositionCurrent and SuperpositionVoltage. These represent signals as a superposition of DC, phasor, transient, and noise signals. They collate the results from the different circuit analysis methods before conversion to the time domain, Laplace domain, *etc*.

All Lcapy expression classes sub-class the Expr class. This facade class provides a common interface to the myriad of Sympy expression classes. Note, Sympy represents an expression using an abstract syntax tree of expression classes (Meurer et al., 2017). For example, it includes simple singleton classes to represent common constants such as 0 or 1, the Mul class to represent the product of two sub-expressions, the Add class to represent the sum of two sub-expressions, *etc*.

The Expr class also tracks the units of an expression using the Sympy units sub-system with an Lcapy extension that simplifies products of units into canonical forms. Units can be printed after expressions in both standard and abbreviated forms.

Lcapy has a Matrix class to extend the Sympy Matrix class. It also has classes to extend list, tuple, and dict. These provide a unified set of methods for printing of expressions.

### Numbers

Lcapy converts floating point numbers to rational numbers of arbitrary precision. This improves detection of equality between expressions and reduces the order of some rational functions.

### Noise signals

Lcapy assumes noise signals are zero-mean, wide-sense stationary, Gaussian random processes. Correlations are specified by a one-sided amplitude spectral density. Each noise source is assigned a noise identifier (nid) attribute. Noise expressions with different nids are assumed to be independent and thus represent different noise realisations.

Lcapy analyses the circuit for each noise realisation independently and stores the result for each realisation separately, using a SuperpositionVoltage or SuperpositionCurrent object. For example:

>>> from lcapy import Circuit

>>> cct = Circuit(" " "

… Vn1 1 0 noise 3

… Vn2 2 1 noise 4" " ")

>>> cct[2].V

$\left\{ {{\rm n1}:3,\;{\rm n2}:4} \right\}.$

In this example Vn1 defines a white Gaussian noise source with an amplitude spectral density (ASD) of 3 V $/\sqrt {{\rm Hz}}$. It has a noise identifier n1. Similarly, Vn2 defines an independent white Gaussian noise source with an ASD of 4 V $/\sqrt {{\rm Hz}}$. It has a noise identifier n2. The voltage at node 2 can be seen to be a superposition of the two noise signals. The total noise ASD at node 2 is found using the n attribute. This sums the noise components in quadrature, *i.e*., $\sqrt {{3^2} + {4^2}}$, since the noise components have different nids and are thus considered independent.

>>> cct[2].V.n

$5.$

### Netlists

Netlists have a Spice-like syntax and are parsed with a custom parser. A parser based on lex-yacc (Johnson, 1975) was tried but found to give poor messages for syntax errors. Each netlist component has an optional orientation hint and drawing attributes. These are used to semi-automatically draw a schematic, using a graph-based approach to define the component positions.

Netlists are stored as ordered dictionaries. This simplifies the lookup of components by name and preserves the component order whenever a netlist is converted into another form.

### Modified nodal analysis

Circuit analysis is performed using modified nodal analysis (MNA) (Ho, Ruehli & Brennan, 1975). Essentially, this analyses the circuit by solving a set of simultaneous linear equations,

(2) $${\bf x} = {{\bf A}^{ - 1}}{\bf b},$$

where $\bf x$ is a vector of unknown nodal voltages and unknown currents through dependent voltage sources, $\bf b$ is a vector of the known voltage and current sources, and **A** is the system matrix. For example, the system of equations for the circuit shown in Fig. 2 and described by the netlist shown in Listing 1 is:

(3) $$\left[ {\matrix{ {{V_1}} \cr {{V_2}} \cr {{I_{{V_s}}}} \cr {} \cr } } \right] = {\left[ {\matrix{ {\displaystyle{1 \over R}}  { - \displaystyle{1 \over R}}  1  \cr { - \displaystyle{1 \over R}}  {\displaystyle{{Ls + R} \over {LRs}}}  0  \cr 1  0  0 \cr } } \right]^{ - 1}}\left[ {\matrix{ 0  \cr 0  \cr {\displaystyle{{{V_s}} \over s}} \cr } } \right].$$

Here *V*_1_ and *V*_2_ are the unknown nodal voltages and *I*_*Vs*_ is the unknown current through the independent voltage source *V*_*s*_. Note, since the voltage source is DC, its Laplace-domain voltage is ${V_s}/s$.

Lcapy uses a custom implementation of modified nodal analysis and supports controlled sources (VCCS, CCVS, *etc*.), opamps, gyrators, mutual inductances, transformers, and two-ports. Each component has a stamp() method that fills in the system matrices.

### Transforms

Lcapy supports the Laplace and the Fourier transform, the discrete-time equivalents (the Z-transform and the discrete-time Fourier transform (DTFT)), plus the discrete Fourier transform (DFT). The inverse transforms are also supported. Where possible, Lcapy breaks an expression into terms and evaluates each term. All results are cached.

#### Laplace transform

Lcapy uses the ${{\rm {\cal L}}_ - }$ form of the Laplace transform^5^,

(4) $${{\rm {\cal L}}_ - }\{ v(t)\} = \int_{{0^ - }}^\infty v(t)\exp ( - st){\rm d}t.$$

Lcapy can evaluate most common Laplace transforms. To improve performance, it caches results. Expressions that Lcapy cannot recognise are passed to Sympy. Unfortunately, Sympy uses the ${\rm {\cal L}}$ form of the Laplace transform,

(5) $${\rm {\cal L}}\{ v(t)\} = \int_0^\infty v(t)\exp ( - st){\rm d}t.$$

However, this only affects Dirac deltas (and their derivatives) and these are handled by Lcapy.

Time-domain expressions are determined using an inverse Laplace transform of the corresponding Laplace-domain expression. The Sympy inverse Laplace transform routines were found to be slow and unreliable and so were augmented with a custom method. This was applied when the expression could be factored into the product of a rational function and an exponential, as described by (1). After finding the poles of the rational function, the residues of the partial fraction expansion are determined using a custom algorithm. When pairs of unrepeated conjugate poles are detected, sines and cosines are generated, otherwise exponential functions (and their derivatives for repeated poles) are generated.

#### Fourier transform

The Fourier transform computed by Lcapy is

(6) $$V(f) = \int_{ - \infty }^\infty v(t)\exp ( - {\rm j}2\pi ft){\rm d}t.$$

Both the forward and inverse Fourier transforms are performed using the same algorithm with a change of variable. Here is an example of usage:

>>> from lcapy import expr, f

>>> v = expr(’sin(2 * pi * f0 * t)’)

>>> v(f)

$- \displaystyle{{{\rm j}\delta \left( {f - {f_0}} \right)} \over 2} + \displaystyle{{{\rm j}\delta \left( {f + {f_0}} \right)} \over 2}.$

The Fourier transform can also be expressed in terms of angular frequency using a change of variable. For example:

>>> V = v(omega)

$- \displaystyle{{{\rm j}\delta \left( { - {f_0} + \displaystyle{\omega \over {2\pi }}} \right)} \over 2} + \displaystyle{{{\rm j}\delta \left( {{f_0} + \displaystyle{\omega \over {2\pi }}} \right)} \over 2}.$

#### Z-transform

The unilateral Z-transform is computed as

(7) $$V(z) = \sum\limits_{n = 0}^\infty v[n]{z^{ - n}}.$$

Lcapy recognises many common discrete-time signals and returns the appropriate Z-transform. For inverse Z-transforms, Lcapy uses partial fraction expansion to simplify expressions.

#### Discrete-time Fourier transform

The DTFT is computed as

(8) $${V_{{1 \over {\Delta t}}}}(f) = \sum\limits_{n = - \infty }^\infty v[n]\exp ({\rm j}2\pi fn\Delta t).$$

The normalized linear and angular frequency forms are handled with a change of variable. For example, the DTFT of the rect function in terms of normalized angular frequency, Ω:

>>> from lcapy import symbol, rect, n, Omega

>>> N = symbol(’N’, even = True)

>>> x = rect(n/N)

>>> x(Omega)

$\displaystyle{{{e^{\displaystyle{{{\rm j}\Omega } \over 2}}}\sin \left( {\displaystyle{{N\Omega } \over 2}} \right)} \over {\sin \left( {\displaystyle{\Omega \over 2}} \right)}},$

and the DTFT of the normalized sinc function in terms of the normalized frequency, *F*:

>>> from lcapy import symbol, sincn, n, F

>>> x = sincn(n * ’K’)

>>> x(F)

$\displaystyle{{\sum_{m = - \infty }^\infty {{\rm rect}} \left[ {\displaystyle{F \over K} - \displaystyle{m \over K}} \right]} \over K}.$

### One-port components

The one-port components are sub-classed from the OnePort class. Parallel and series combinations are represented by the Par and Ser classes. Both of these combinations are also OnePort objects.

### Two-port components

Two-port components are based on the TwoPortModel class. This class contains a 2 × 2 matrix of expressions for the two-port parameters. This is augmented with a vector of two voltages, two currents, or a voltage/current pair to describe the effects of independent sources within the circuit. For example, the equations describing a B-parameter two-port model are:

(9) $$\left[ {\matrix{ {{V_2}} \cr { - {I_2}} \cr } } \right] = \left[ {\matrix{ {{B_{11}}} & {{B_{12}}} \cr {{B_{21}}} & {{B_{22}}} \cr } } \right]\left[ {\matrix{ {{V_1}} \cr {{I_1}} \cr } } \right] + \left[ {\matrix{ {{V_{2b}}} \cr {{I_{2b}}} \cr } } \right].$$

Here *V*_2*b*_ and *I*_2*b*_ model the independent sources within the circuit. Similarly, the equations used to describe a Z-parameter two-port model are:

(10) $$\left[ {\matrix{ {{V_1}} \cr {{V_2}} \cr } } \right] = \left[ {\matrix{ {{Z_{11}}} & {{Z_{12}}} \cr {{Z_{21}}} & {{Z_{22}}} \cr } } \right]\left[ {\matrix{ {{I_1}} \cr {{I_2}} \cr } } \right] + \left[ {\matrix{ {{V_{1z}}} \cr {{V_{2z}}} \cr } } \right].$$

**Figure 6:**
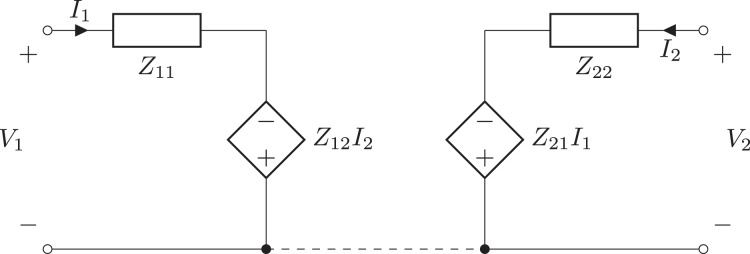
Independent source-free Z-parameter two-port model.

**Figure 7:**
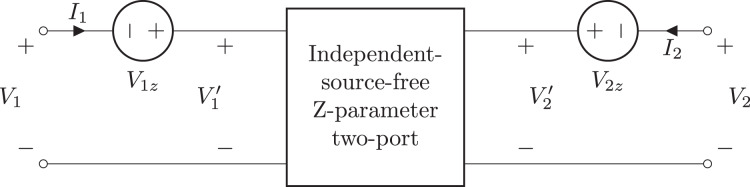
General Z-parameter two-port model.

In this case, a pair of voltages (one for each port) are required to model the independent sources within the circuit, see Figs. 6 and 7.

### Nodal and mesh analysis

**Figure 8:**
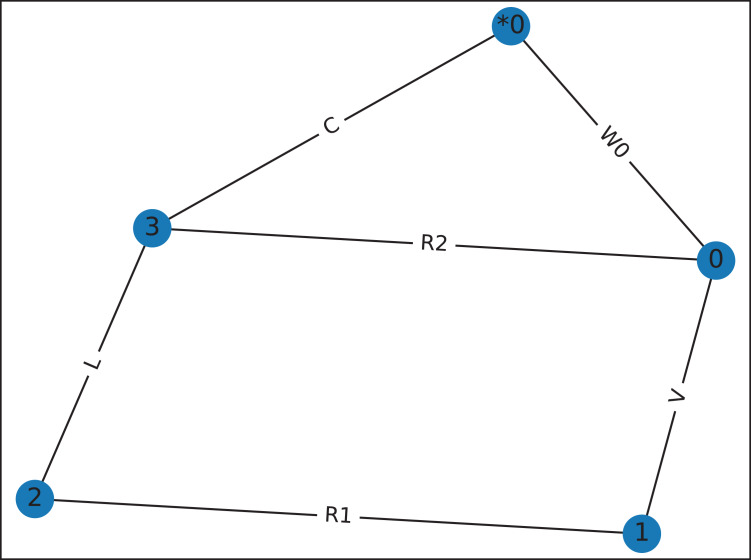
Graph for Fig. 4, drawn by graphviz, showing the dummy wire, W0, added to avoid the parallel connection between R2 and C. This graph has two meshes for mesh-analysis.

Nodal and mesh analysis use the CircuitGraph class to convert a netlist into a graph. The Python package Networkx (Hagberg, Swart & Chult, 2008) is employed to determine the loops in the graph. Unfortunately, Networkx cannot find loops when there are parallel edges in a graph, and so Lcapy adds dummy nodes and dummy wires to avoid parallel edges (see Fig. 8).

## Performance

The performance of Lcapy is strongly dependent on the matrix inversion algorithm used by Sympy. This constrains the complexity of the circuit that can be solved interactively.

**Figure 9:**
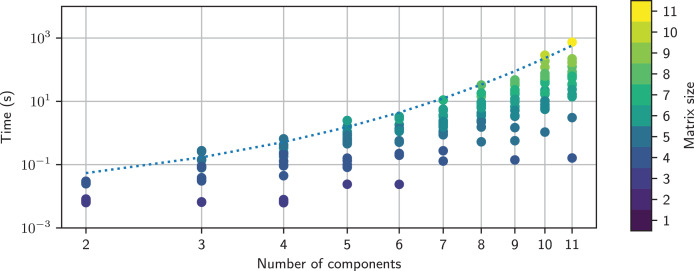
Timing comparison for networks comprised of a number of resistors and a single voltage source. This is colored by the MNA matrix size; this is larger for series connections than for parallel connections. An empirical model for the maximum time is shown as the dashed line. The MNA matrix was inverted using the adjoint matrix approach.

Figure 9 shows the time taken to determine the open-circuit voltage for twenty randomly-generated networks with a specified number of components. Each network has a single voltage source and a number of resistors, and its open-circuit voltage was computed using:

>>> net = random_network(num_resistors = NR, num_voltage_sources = 1, kind = ‘dc’)

>>> net.Voc

**Figure 10:**
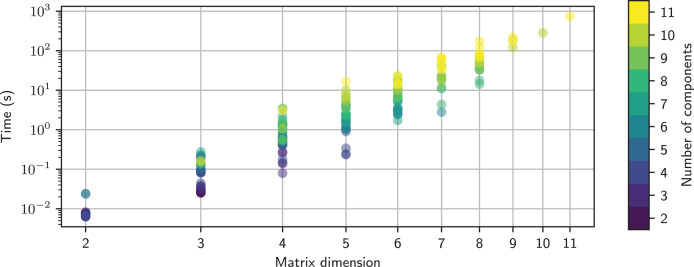
Timing comparison for networks comprised of a number of resistors and a single voltage source. This is plotted as function of the MNA matrix size and is colored by the number of components in each random network. The MNA matrix was inverted using the adjoint matrix approach. Note, the size of the MNA matrix is larger for series connections than for parallel connections.

The timing tests were performed on an HP Elitebook 850 G6 using a single core of an Intel i5-8365U CPU running at 1.60 GHz. It is apparent that the maximum computation time increases exponentially with the number of components. The computation time is also highly variable for a given number of components, with over three orders of magnitude variation for 11 components. This is partly due to the MNA matrix size, but it also depends on the number of terms that each matrix element has. The variation of time with the MNA matrix size is not obvious, as shown in Fig. 10.

**Figure 11:**
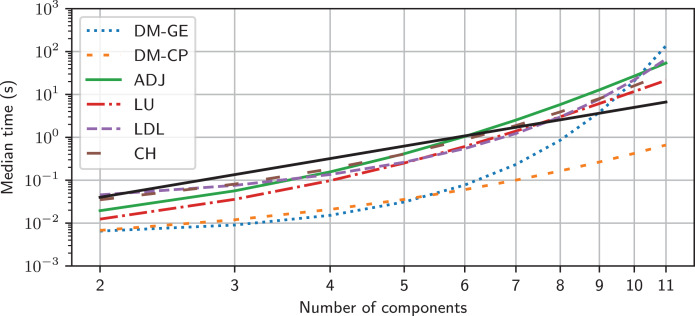
Empirical models for the maximum time taken to invert the MNA matrix as a function of number of components in a random network using different inversion methods. The solid black line represents *O*(*N*^3^). DM-GE denotes Gaussian elimination using Domain Matrix DM-CP denotes the characteristic polynomial method using Domain Matrix.

Sympy has a number of matrix inversion algorithms. Historically, Lcapy used Gaussian elimination since it was fastest, however, the latest version of Sympy has disappointing performance for some of the MNA matrices generated by Lcapy. The other methods produce a similar maximum time, as shown in Fig. 11. The exception is the new domain matrix (DM) algorithm, currently under development, which shows promising results. These algorithms are *O*(*N*^3^) as expected (Krishnamoorthy & Menon, 2013) but only for small *N* × *N* matrix sizes. For larger *N*, the computation time is slower due to the additional arithmetic required to maintain all the terms.

### Documentation

Documentation is stored as Restructured Text and converted to HTML/PDF using Sphinx (http://sphinx-doc.org/sphinx.pdf). On-line documentation can be found at https://lcapy.readthedocs.io. Included in the documentation are tutorials; some using Jupyter notebooks.

## Discussion

Lcapy leverages from the strengths of Python as an interpreted programming language and Sympy as a computer algebra system. Its advantage over other circuit analysis programs is that it can generate and solve systems of integro-differential equations. The symbolic solutions explicitly shows how component values affects circuit behaviour and allows an exact solution to be calculated. A unique feature of Lcapy is that it can perform noise analysis symbolically.

Lcapy can present the circuit equations for nodal analysis, modified nodal analysis, loop analysis, and state-space analysis. Expressions can be represented in many different formats, parameterized, and transformed between domains, such as Laplace and Fourier. Expressions can be associated with a quantity to ensure correct dimensional calculations and to print expressions with units.

Textbook-quality schematics can be generated in a number of formats from a network or netlist description. These can be customised for different conventions.

Symbolic analysis provides an exact solution and avoids numerical inaccuracies and instabilities. However, it is much slower for large problems. The time-critical functions are matrix inversion for modified nodal analysis and pole/zero finding. The computation time could be improved using the Simplification Before Generation (SBG) approach (Kolka, Biolek & Biolkova, 2008; Kolka, Vlk & Horak, 2011). To mitigate slow computation, wherever possible Lcapy uses lazy evaluation and caches results.

Sympy is an excellent tool but can be confusing for novices since it allows symbols of the same name but with different assumptions. Thus the symbols are not equivalent. Lcapy tries to resolve this problem by keeping a registry of symbols and looking them up by name. The disadvantage of this approach is that symbol assumptions cannot be changed.

Lcapy has a number of circuit transformations but further work could add more advanced circuit transformations, similar to programs like SAPWIN (Luchetta, Manetti & Reatti, 2001). Another improvement for circuit analysis teaching would be to incorporate a trainer program (Weyten, Rombouts & De Maeyer, 2009).

## Conclusions and future work

Lcapy is an open-source Python package, designed to generate and solve systems of equations for linear circuit analysis. It supports many aspects of circuit theory, including two-port networks, network synthesis, and polyphase systems. The author is aware of its use for generating exam questions, checking exam and homework answers, teaching students circuit theory and signal processing, simulating polyphase power systems, designing controllers for power-electronics, modelling low-noise operational amplifier circuits, and modelling electromechanical systems. There are many other potential applications.

Planned work is to improve compatibility with PySpice and to add a graphical user interface (GUI) to enter and display circuits schematically. This could be achieved by importing and exporting netlists or implementing the GUI in Python and using Lcapy as a module. A GUI is useful from a circuit teaching perspective since components could be selected, the current/voltage interrogated and specific transforms applied.

An improvement would be to employ cancellation-free analysis so that all poles and zeros are shown. One approach is to use graph-pair decision diagram techniques (Gu & Shi, 2015) or heuristic tree-based methods (Fernández, Rodríguez-Vázquez & Huertas, 1990). Another improvement would be to use heuristics to generate approximate solutions (Yu & Sechen, 1996; Fernández et al., 1994; Wambacq et al., 1995; Wambacq, Gielen & Sansen, 1998; Katzenelson & Unikovski, 1999). These enhancements to Lcapy could be easily added by switching the MNA solver class. Another improvement would be the linearisation of non-linear circuits around an operating point for small-signal analysis.

## Footnotes

1 The three greater-than signs denote the user input for the Python interactive session.

2 All the expressions in this paper are presented using the latex method.

3 Lcapy is open-source and hosted on GitHub at https://github.com/mph-/lcapy. It is distributed under the GNU Lesser General Public License v2.

4 The expression classes are for internal use and are not part of the the API.

5 As advocated by Lundberg, Miller & Trumper (2007).

## Additional Information and Declarations

### Data Availability

The following information was supplied regarding data availability: The code is available at GitHub: https://github.com/mph-/lcapy.

## References

[ref-1] Bakshi A, Bakshi U (2005). Network analysis and synthesis.

[ref-2] Biolek D (2000). SNAP-program with symbolic core for educational purposes.

[ref-3] Brinson M, Jahn S (2009). Qucs: a GPL software package for circuit simulation, compact device modelling and circuit macromodelling from DC to RF and beyond. International Journal of Numerical Modelling: Electronic Networks, Devices and Fields.

[ref-4] Cervone D (2012). Mathjax: a platform for mathematics on the web. Notices of the American Mathematical Society.

[ref-5] Chen W, Shi G (2006). Implementation of a symbolic circuit simulator for topological network analysis.

[ref-6] Fernández F, Rodríguez-Vázquez A, Huertas J (1990). ASAP: a portable program for the symbolic analysis of analog integrated circuits.

[ref-7] Fernández FV, Rodríguez-Vázquez A (1996). Symbolic analysis tools-the state of the art.

[ref-8] Fernández FV, Wambacq P, Gielen G, Rodríguez-Vázquez A, Sansen W (1994). Symbolic analysis of large analog integrated circuits by approximation during expression generation.

[ref-9] Gielen GG, Walscharts HC, Sansen WM (1989). ISAAC: a symbolic simulator for analog integrated circuits. IEEE Journal of Solid-State Circuits.

[ref-10] Grasso F, Luchetta A, Manetti S, Piccirilli M, Reatti A (2014). SapWin 4.0 an enhanced tool for analysis and design of analog circuits.

[ref-11] Gu Y, Shi G (2015). An interactive program for automatic network function generation with insights.

[ref-12] Hagberg A, Swart P, Chult S, D (2008). Exploring network structure, dynamics, and function using NetworkX.

[ref-13] Hayes MP (2014). Lcapy: Linear circuit analysis with Python.

[ref-14] Hayes MP (2016). Semi-automated electrical circuit drawing with Lcapy.

[ref-15] Hayes MP (2020). Lcapy’s documentation. https://lcapy.readthedocs.io.

[ref-16] Hayt WH, Kemmerly JE, Durbin SM (2006). Engineering circuit analysis.

[ref-17] Heffernan B, Mitchell B, Hayes M (2020). Trapezoidal current generator for an electromagnetic groundwater flowmeter.

[ref-18] Ho C-W, Ruehli AE, Brennan PA (1975). The modified nodal approach to network analysis. IEEE Transactions on Circuits and Systems.

[ref-19] Hsu J-J, Sechen C (1993). Low-frequency symbolic analysis of large analog integrated circuits.

[ref-20] Hsu J-J, Sechen C (1994). Fully symbolic analysis of large analog integrated circuits.

[ref-21] Huelsman LP (1996). Symbolic analysis-a tool for teaching undergraduate circuit theory. IEEE Transactions on Education.

[ref-22] Hunter JD (2007). Matplotlib: a 2D graphics environment. Computing in Science & Engineering.

[ref-23] Johnson SC (1975). Yacc: yet another compiler-compiler.

[ref-24] Joyner D, Čertík O, Meurer A, Granger BE (2012). Open source computer algebra systems: SymPy. ACM Communications in Computer Algebra.

[ref-25] Katzenelson J, Unikovski A (1999). Symbolic-numeric circuit analysis or symbolic circuit analysis with online approximations. IEEE Transactions on Circuits and Systems I: Fundamental Theory and Applications.

[ref-26] Khanshan AH (2007). Electrical two-ports; a Maple approach.

[ref-27] Kluyver T, Ragan-Kelley B, Pérez F, Granger BE, Bussonnier M, Frederic J, Kelley K, Hamrick JB, Grout J, Corlay S (2016). Jupyter notebooks-a publishing format for reproducible computational workflows.

[ref-28] Kolka Z, Biolek D, Biolkova V (2008). Symbolic analysis of linear circuits with modern active elements. WSEAS Transactions on Electronics.

[ref-29] Kolka Z, Vlk M, Horak M (2011). Topology reduction for approximate symbolic analysis. Radioengineering.

[ref-30] Krishnamoorthy A, Menon D (2013). Matrix inversion using Cholesky decomposition.

[ref-31] Luchetta A, Manetti S, Reatti A (2001). SAPWIN-a symbolic simulator as a support in electrical engineering education. IEEE Transactions on Education.

[ref-32] Lundberg KH, Miller HR, Trumper DL (2007). Initial conditions, generalized functions, and the Laplace transform troubles at the origin. IEEE Control Systems Magazine.

[ref-33] Meurer A, Smith CP, Paprocki M, Čertík O, Kirpichev SB, Rocklin M, Kumar A, Ivanov S, Moore JK, Singh S, Rathnayake T, Vig S, Granger BE, Muller RP, Bonazzi F, Gupta H, Vats S, Johansson F, Pedregosa F, Curry MJ, Terrel AR, Roučka V, Saboo A, Fernando I, Kulal S, Cimrman R, Scopatz A (2017). Sympy: symbolic computing in Python. PeerJ Computer Science.

[ref-34] Nagel LW, Pederson DO (1973). SPICE: Simulation program with integrated circuit emphasis.

[ref-35] Oliphant TE (2006). A guide to NumPy.

[ref-36] Pérez F, Granger BE (2007). IPython: a system for interactive scientific computing. Computing in Science & Engineering.

[ref-37] Shi G (2017). Topological approach to symbolic pole-zero extraction incorporating design knowledge. IEEE Transactions on Computer-Aided Design of Integrated Circuits and Systems.

[ref-38] Srivastava A, Wierzba G, MacKay J (1990). Symbolic approximation of analog circuits using Sspice.

[ref-39] Tlelo-Cuautle E, Quintanar-Ramos A, Gutiérrez-Pérez G, de la Rosa MG (2004). SIASCA: interactive system for the symbolic analysis of analog circuits. IEICE Electronics Express.

[ref-40] Ushie OJ, Abbod M, Ashigwuike E (2015). MATLAB symbolic circuit analysis and simulation tool using PSpice netlist for circuits optimization. International Journal of Engineering and Technology Innovation.

[ref-41] van der Walt S, Colbert SC, Varoquaux G (2011). The NumPy array: a structure for efficient numerical computation. Computing in Science & Engineering.

[ref-42] van Rossum G, Drake FL (1995). Python reference manual.

[ref-43] Vazzana GA, Grasso AD, Pennisi S (2017). A toolbox for the symbolic analysis and simulation of linear analog circuits.

[ref-44] Virtanen P, Gommers R, Oliphant TE, Haberland M, Reddy T, Cournapeau D, Burovski E, Peterson P, Weckesser W, Bright J (2020). SciPy 1.0: fundamental algorithms for scientific computing in Python. Nature Methods.

[ref-45] Walscharts H, Gielen G, Sansen W (1989). Symbolic simulation of analog circuits in s-and z-domain.

[ref-46] Wambacq P, Fernández F, Gielen G, Sansen W, Rodrguez-Vázquez A (1995). Efficient symbolic computation of approximated small-signal characteristics of analog integrated circuits. IEEE Journal of Solid-State Circuits.

[ref-47] Wambacq P, Gielen G, Sansen W (1998). Symbolic network analysis methods for practical analog integrated circuits: a survey. IEEE Transactions on Circuits and Systems II: Analog and Digital Signal Processing.

[ref-48] Weyten L, Rombouts P, De Maeyer J (2009). Web-based trainer for electrical circuit analysis. IEEE Transactions on Education.

[ref-49] Wierzba G, Srivastava A, Joshi V, Noren K, Svoboda J (1989). Sspice-a symbolic spice program for linear active circuits.

[ref-50] Yu Q, Sechen C (1996). Approximate symbolic analysis of large analog integrated circuits. IEEE Transactions on circuits and systems-1. Fundamental theory and applications.

